# Children’s preferences for features and designs of KN95-style respirators: A comparative study between Indonesia and Nepal

**DOI:** 10.1371/journal.pone.0334116

**Published:** 2025-10-17

**Authors:** Sarah Nila, Chandika Shrestha, Hastin M. Maharti, Claire J. Horwell, Dicky C. Pelupessy, Rachel L. Kendal, Meghnath Dhimal, Judith Covey

**Affiliations:** 1 Department of Psychology, Durham University, Durham, United Kingdom; 2 Department of Anthropology, Durham University, Durham, United Kingdom; 3 Faculty of Psychology, Universitas Indonesia, Depok, Indonesia; 4 Institute of Hazard, Risk & Resilience, Department of Earth Sciences, Durham University, Durham, United Kingdom; 5 Nepal Health Research Council, Kathmandu, Nepal; Universitas Syiah Kuala, INDONESIA

## Abstract

Air pollution is a global crisis, posing significant health risks to humans. Children are particularly vulnerable to the effects of particulate air pollution, and the use of respiratory protection could reduce their exposure. Certified, well-fitting respirators have been shown to effectively filter airborne particles and are increasingly available for children in non-occupational settings. However, their effectiveness relies on proper fit and consistent use. Understanding children’s perspectives on wearing respiratory protection, specifically their preferences for different types and styles of respirators, is crucial. To explore these issues, 12 focus group discussions were conducted in January 2023 with 116 children aged 6–12 years living in Kathmandu, Nepal (N = 67) and Bandung, Indonesia (N = 49). Participants were recruited from public and private schools in each city using purposive sampling to ensure diversity in age, gender and socio-economic background. Focus groups were held in school settings and facilitated by local researchers in the children’s native languages. Children were shown seven different respirators and asked questions about their preferred styles and features. Statistical analyses using Wilcoxon one-sample tests and ordinal regression showed the most popular types of respirators had ear-loops rather than head-straps and some features (e.g., patterned rather than plain masks) were more popular with certain groups (e.g., younger children). These results suggest that respirator manufacturers should ideally offer a variety of styles or designs.

## Introduction

Air pollution has emerged as a critical global issue, with airborne concentrations of ambient pollutants frequently surpassing regulatory thresholds and posing significant health risks for both acute and chronic conditions [1,2], including acute respiratory infections [3]. This complex mixture of gases, aerosols, and particulate matter (PM) varies across different micro-environments such as outdoor spaces, homes, and workplaces. Because of its extensive negative impacts on human health, air pollution remains one of the most significant challenges to achieving the good health and well-being Sustainable Development Goal (SDG) adopted by the United Nations Member States in 2015 [4]. Children are particularly at risk from inhaling air pollution, with cumulative exposure affecting children into adulthood, causing a range of diseases that will risk quality of life, earning potential and societal development [5].

Research has suggested that individuals can reduce their exposure to air pollutants by taking actions such as avoiding physical activities near high-traffic roadways or combustion sources and by staying indoors with closed windows [6]. However, these measures may not be adequate for individuals who must spend time outdoors for work or school, and keeping windows closed may not be feasible in tropical regions and potentially traps indoor pollutants.

Face coverings which cover the nose and mouth offer a potential personal protective measure against air pollution for both adults and children. However, commonly available and inexpensive cloth and surgical masks have been found to have poor particle filtration efficiency and inward leakage due to their materials and fit [7–10]. In contrast, certified respirators, tested to particulate filtration standards such as N95/99 (US), FFP2/3 (EU/UK), KN95 (China), and KF94 (Korea), have demonstrated effectiveness in filtering air pollution particles and other airborne particles such as virus-laden micro-droplets (including SARS-CoV-2), and volcanic ash [9–14]. The efficacy of certified respirators also hinges on their fit to the face [10,15–18].

While most studies have focused on adult populations, research indicates that respirators can also be effective and safe for children to use, if they can be made to fit properly [16,19]. Policymakers must, however, consider more than just the efficacy, safety and cost of respirators for children; they must also account for the likelihood of children wearing them, which may depend on their experiences and perceptions of mask-wearing as well as their preference for different styles of masks. The present study focuses on children’s perceptions and preferences of different designs of respirators.

Two recently published studies have underscored the importance of mask/ respirator appearance and design for children [20,21]. Smart et al. [21] examined the wearability of three children’s respirators marketed in the UK. Children aged 8–11 years from London, pre-pandemic, rated the three respirators for appearance, and comfort, hotness, breathability, and fit after standardized walking and running activities. Respirators with a nose clip received the highest ratings for perceived fit, while a respirator with a green colored and patterned front layer scored highest ratings for appearance. To supplement a systematic review of children’s experiences of mask-wearing (which included findings from Smart et al. [21]), Preest et al. [20] conducted an online consultation with children from the UK. Nine children between the ages of 6 and 13 years submitted drawings of their ideal masks. Thematic analysis of the drawings revealed that mask appearance was important to the children and indicated that the younger children in the sample preferred playful, colorful, and decorative masks, while the older children valued uniformity (i.e., simple designs that everyone can wear) in school settings.

Although these studies suggest that younger children are more likely to wear masks with appealing patterns and colors, the findings are based on small convenience samples from the UK and should not be generalized. The UK is also a high-income country (HIC), where air pollution levels are generally lower than in low- or middle-income countries (LMICs) [22]. Public health infrastructure is stronger, and mask-wearing norms might also be different than in other countries. Obtaining perspectives from diverse countries and cultures, especially from children in LMICs facing high levels of particulate air pollution on a daily basis, is crucial in working towards the UN SDGs.

To address this gap, we examined the respirator design preferences of children aged 6–12 years from two cities: Kathmandu, Nepal and Bandung, Indonesia. Schools in these two locations were participating in a research project on protecting children from air pollution (Factors Affecting Childhood Exposures to Urban Particulates; FACE-UP https://face-up-consortium.webspace.durham.ac.uk/). The study presented here is one work package from the FACE-UP project. These locations were chosen for the FACE-UP project because of their severe air pollution issues and because they are both eligible for Overseas Development Assistance (ODA). In 2023, both countries had average annual PM_2.5_ concentrations exceeding WHO guidelines by 7–10 times [23]. The cities also have comparable population densities (in excess of 15,000 people per km^2^) and topographies (bowl-shaped basins which trap and accumulate pollutants) [24,25].

Respirators can only be effective as an intervention if children are willing to wear them, so we collected quantitative data to assess how children’s willingness to wear respirators was influenced by four key design features – pattern, color, shape, and strap design. We examined how children’s preferences for these features varied between the two countries and by age, gender and socio-demographic characteristics. The distinct cultural, demographic, socioeconomic, climatic, and urban characteristics of these cities make our comparison valuable for considering whether city-specific strategies are needed to promote mask-wearing among children.

Although our primary objective was to identify children’s preferences for different designs of respirators, we also explored their general perspectives on mask-wearing through focus group discussions. We were interested, for instance, in whether the children in Nepal and Indonesia would highlight the same issues (such as discomfort, breathing difficulties, heat inside the mask, and headaches) identified by children in previous research [20,21,26,27]. These insights can help governmental agencies and related organizations develop strategies to encourage consistent mask-wearing especially during air pollution crises which was a core purpose of the FACE-UP.

## Methods

### Participants

The research was conducted in Kathmandu, Nepal and Bandung, Indonesia between January 24^th^ and February 1^st^, 2023. The research protocol used in this study was approved by the ethics committees from the Department of Psychology, Durham University (Ref: PSYCH-2022-06-01T12_54_54-dps0jac), the Nepal Research Health Council (NHRC) (Ref: 502/2022), and the Faculty of Psychology, Universitas Indonesia (Ref: 181/FPsi.Komite Etik/PDP.04.00/2022). Parents were provided with a written information sheet about the project and provided signed consent for their child to take part in the project. The parents were also provided with a simplified information sheet to read out to their child. As a requirement of the NHRC ethics committee the Nepalese children also signed an assent form. Written assent of the children was not required by the Universitas Indonesia ethics committee. All the children provided verbal assent to take part at the beginning of the focus groups. Additional information regarding the ethical, cultural, and scientific considerations specific to inclusivity in global research is included in the Supporting Information (S4 File). All parent- and child-facing documents were translated into the local languages. To check for accuracy all translated documents were independently back translated into English.

From the start of January 2023, we recruited children using a purposive sampling procedure to ensure a mix of children across different grades/ ages, genders, and socio-economic groups. Socio-economic variation was achieved by recruiting children from two schools in each city which represented different socio-economic groups – one public school (a school funded by the government) and one private school (a school funded by tuition fees paid directly to the institution, typically by the child’s guardian). This recruitment method was designed to ensure that our sample represented the socio-demographic diversity within each city and avoided over-representing any particular groups.

From each school, with the support of the teachers and their parents, we recruited children to take part in a focus group discussion (FGD). Three FGDs took part in each school, and we aimed to recruit 10–12 children with equal numbers of boys and girls. There were no exclusion criteria. These targets were met in the Nepalese sample where an average of around 11 children took part in each FGD (N = 67). However, in the Indonesian sample we only recruited an average of eight children for each FGD (N = 49) because attendance at some groups was affected by heavy rain, and it was not possible to reschedule due to constraints imposed by the schools. We inadvertently recruited three 6-year-olds into the FGDs groups (our intended age range was 7–12 years). This was due to the teachers selecting children whom they thought were 7 years old to take part and the children’s actual age was not apparent until after the FGDs had taken place. We informed the Durham University ethics committee of this oversight, and they confirmed that the data for the 6-year-old children could be used in our analyses.

### Materials and procedure

Each selected child was given a consent form and information sheet to take home to their carers to ensure that they were fully informed about this study and had the opportunity to consider whether they wanted their child to participate. In addition to the consent form and information sheet, several other documents were included, such as an assent form for the child (Nepal only, due to government regulations), a privacy notice, and a demographic questionnaire. The demographic questionnaire asked carers to provide their age in years, gender, highest education level, religion, and net monthly income. The questionnaire also included questions about the age and gender of the child who was taking part in the study.

The FGDs were conducted in the local language. At the beginning of each FGD, when the children’s assent had been confirmed, the researchers explained the purpose of the research, the activities that would be conducted, and that the sessions would be audio recorded. The children were assured that they could leave the sessions at any time without providing a reason. We ensured that the FGDs took place in a welcoming and non-threatening environment. Before the discussions, our local researchers emphasized that there were no right or wrong answers, encouraging openness and honesty. Although the discussions were audio-recorded, we reassured the children that their responses would remain confidential, and their identities would not be disclosed. This reassurance aimed to alleviate any concerns about peer judgment and encourage them to share their genuine preferences and experiences with mask-wearing.

**Fig 1:**
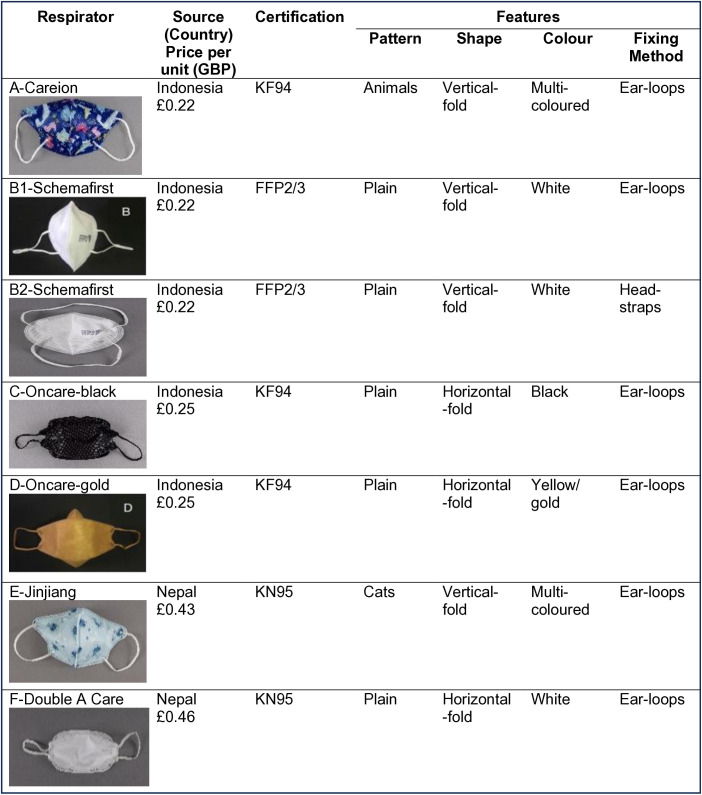
Selected respirators that were used for the feature preference rating and ranking task.

In each session, the children were shown seven different respirators that had been sourced from online retailers or pharmacies in Nepal and Indonesia (see Fig 1). All the respirators were marketed for children (but not for babies) and stated on the packaging that they were certified to a classification (e.g., KN95, KF94, FFP2/3) and sometimes had a standard (e.g., GB2626−2019 for KN95) printed on the packaging and/or respirator itself. The respirators were chosen to represent a variety of different patterns, colors, shapes, and strap designs.

The children were asked to provide their preferences for different features. They were shown examples of each feature from the seven respirators. Each child was given a printed chart, and they chose between patterned or plain, ear-loops or head-straps, vertical-fold or horizontal-fold design, colored or white, black or white, and black or colored by ticking a box for each of their preferences. For all comparisons they could indicate no preference (do not mind).

It is noted that some of the same feature preference questions were included in a different work package that was conducted under the auspices of the FACE-UP project (https://face-up-consortium.webspace.durham.ac.uk/). In this study, which was conducted about five months after our one, a smaller sample of children from Nepal and Indonesia were asked whether they preferred colored vs. white, vertical-fold vs. horizontal-fold and (in the Nepal sample only) patterned vs. plain respirators after they had worn a number of different respirators for fit and wearability tests [19]. The differences found between the sample percentages in the two studies were not big enough to be statistically significant.

Once the feature preference activity was completed, the children were asked to provide a ranking of six of the respirators. Respirator B2, the only respirator with a head-strap, was excluded from the ranking activity; B1 was the same respirator but with ear-loops. This was done so that all respirators had ear-loops, so children’s rankings were only related to mask shape, pattern and color. Each child was given a ranking chart and laminated images of each of the respirators. They were then asked to place the laminated cards on the chart, in the order of their preference, with their most favourite at the top and least favourite at the bottom. The children were told that they could place more than one card in the same rank if they liked the respirators equally.

A general discussion was also held in the FGDs to explore the reasons why children wore masks and their perspectives on mask wearing. We inquired: (1) Whether they had previously worn a mask for general purposes, (2) Whether they had started wearing masks before or during the COVID-19 pandemic, (3) The reason why they wore masks aside from protection against the SARS-COV-2 virus, (4) Their thoughts on wearing masks, including their willingness to wear one and their perceptions of comfort while doing so, and (5) For those who did not feel comfortable, we asked them to elaborate on the reasons, including both physical and psychological factors.

At the end of the FGD, each child was given a small gift as an appreciation for their time and participation. In Nepal, younger children were given a lunch box and older children a maths geometric set box. In Indonesia, all children were given a lunch box.

### Data analysis

The ranking task was scored with values from 1 to 6 assigned to the respirators ranked in first to sixth place. In the case of tied ranks the respirators received the same average rank. For example, if two respirators were ranked first then they were both assigned a rank of 1.5. A Friedman test was undertaken to test for differences in the median ranks among the six masks. If the result was significant, post hoc Wilcoxon tests with Bonferroni correction for multiple comparisons were conducted to locate which pairs of respirators were ranked significantly differently from each other. Although Bonferroni correction is conservative and may increase the risk of Type II errors, it was chosen for its strict control of family-wise error rate. This conservative approach was deemed appropriate given the exploratory nature of the comparisons and the need to minimize false positives (Type I errors).

The preference ratings for each feature (e.g., patterned vs. plain) were scored on a 3-point ordinal scale: e.g., + 1 (prefer patterned), 0 (do not mind), and −1 (prefer plain). Descriptive analysis was conducted to show the frequency and percentage of the feature preferences. Two-tailed Wilcoxon one-sample tests were conducted to determine whether feature preference ratings differed significantly from zero (0 = do not mind). These analyses were conducted by S.N. using R software version 4.2.2.

Ordinal regression analyses were conducted to explore whether the country which the children came from and demographic variables (i.e., children’s gender, age, and socio-economic status) were predictive of their feature preferences. These analyses were conducted by J.C. using IBM SPSS version 27.

The FGD discussions were analysed in the local languages by C.S and H.M using thematic analysis [28] which facilitated the identification, analysis, and interpretation of key themes within the qualitative data. This method provided a comprehensive understanding of children’s experiences and perspectives. The full lists of codes and themes/ sub-themes can be found in S1 and S2 Tables. The codes, themes, and all quotes included in this paper were translated from the local languages into English by C.S. and H.M. Translations were checked by one of the research assistants K.S. (see Acknowledgments) and S.N.

## Results

### Demographic characteristics

The demographic characteristics of the children who took part in the FGDs are reported in S3 Table.

In Nepal, out of the 67 children who took part, 34 were recruited from the public school and 33 from the private school, 37 (55.2%) of them were boys and the mean age was 9.75 years (SD = 1.71, Min = 6.00, Max = 12.92). 52.2% of the carers who provided consent were women with a mean age of 32.9 years (SD = 8.14). 58.2% of carers were educated to at least secondary/ higher secondary level (grade 10−12) and 7.5% of carers had completed a bachelor’s or postgraduate degree. The mean monthly household income was 36,045 NPR or equivalent to 267.1 USD (SE = 4742 NPR/ 35 USD, November 2024 rates), although 34.3% of carers did not provide their income. This mean monthly household income is about 20% lower than the mean for Nepal (45,929 NPR) as reported in the Nepal Living Standards Survey in 2022−2023, although the upper limit of the 95% confidence interval of the mean (i.e., M + 1.96 SE = 45,339 NPR) is within 590 NPR (4.36 USD) of the Nepal mean [29]. It is notable that the carers’ education levels and household incomes were not significantly related to the child attending private school (education levels r_s_ ≤ .176, *P* ≥ .221; household income r_s_ = −.131, *P* = .395). This suggests that the type of school the child was attending may not be a good indicator of socio-economic status in the Nepalese sample.

In Indonesia, out of the 49 children who took part, 25 were recruited from the public school and 24 from the private school, 25 (51.0%) of them were boys and the mean age mean was 9.46 years (SD = 1.60, Min = 6.37, Max = 12.83). Most of the carers were women (85.7%) with a mean age of 38.4 years (SD = 5.95). 97.9% of the carers were educated to at least secondary/ higher secondary level (grade 10–12) and 55.1% of carers had completed a bachelor’s or postgraduate degree. The median monthly household income was in the 2–5 million IDR band or equivalent to 127.7–379.2 USD (November 2024 rates), although 28.6% of carers did not provide their income. This band includes the average for monthly per capita earnings in Indonesia reported by CEIC [30]. In the Indonesian sample, the carers’ education levels were positively related to the child attending private school (education levels rs ≥ .715, *P* < .001), although the relationship between household income and attending private school was not quite significant (r_s_ = .325, *P* = .057). This suggests that the type of school the child was attending is, at most, a weak indicator of socio-economic status in the Indonesian sample.

### Ranking and feature preference ratings

As shown in Table 1, respirators A (Careion), C (Oncare-black), and E (Jinjiang) were more frequently ranked first, with median ranks of 3 putting them in the top half of the ranking for most children. There was, however, no clear favorite among the six respirators. A Friedman test was conducted to test for differences in the median ranks among the six respirators. This revealed a significant difference in the median ranks of the six respirators (Χ^2^ (5) = 26.5, *P < .001*). Post hoc Wilcoxon tests with Bonferroni correction for multiple comparisons were conducted to locate which pairs of respirators were ranked significantly differently from each other. These post hoc tests indicated that respirator D, which was a yellow/ gold color, was ranked significantly lower than respirators A (Careion), C (Oncare-black), E (Jinjiang), and F (Double A Care). Only 4.5% of children ranked this respirator first, with a median rank of 4.

**Table 1:** Frequency (N) and percentage (%) of children in the full sample who ranked each respirator first and last.

Respirator	Ranked first N (%)	Ranked last N (%)	Median rank^i^
A (Careion)	42 (26.8)	27 (19.4)	3^a^
B1 (Schemafirst)	20 (12.7)	17 (12.2)	3.75
C (Oncare-black)	32 (20.4)	12 (8.6)	3^b^
D (Oncare-colored)	7 (4.5)	40 (28.8)	4^a, b, c, d^
E (Jinjiang)	35 (22.3)	24 (17.3)	3^c^
F (Double A Care)	21 (13.4)	19 (13.7)	3.5^d^

^i^First rank = 1, Last rank = 6. A Friedman test showed a significant difference in the median ranks assigned to the six respirators (Χ^2^ (5) = 26.5, **P* *< .001**). Post hoc comparisons with Bonferroni adjustment found significant differences between the respirators with the same superscripts.

**Fig 2:**
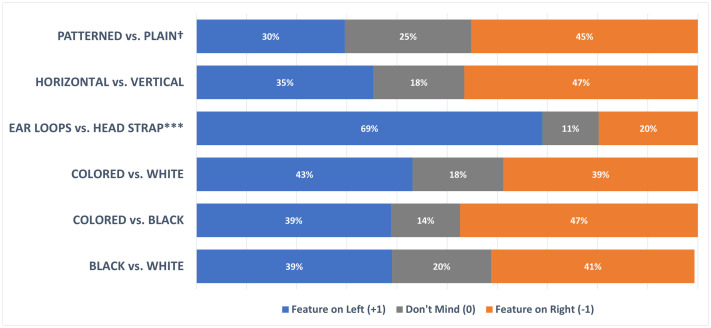
Feature preferences in the full sample. Significance values shown are based on the results of one-sample Wilcoxon tests for which the median rating was significantly different from zero. ^†^*P* < .10, *** **P* *< .001.

Fig 2 displays the distribution of feature preference ratings in the full sample showing the percentage of children who preferred either the feature on the left (e.g., patterned), right (e.g., plain) or did not have a preference for either feature (don’t mind). It is notable that for most of the comparisons, the majority of children had a preference for one or other feature. The percentage of children who did not have a preference was at most 25% (patterned vs. plain). That being said, the children who did have a preference were quite evenly split between the features they were comparing. Wilcoxon one-sample tests were conducted to determine whether feature preference ratings differed significantly from zero (0 = do not mind). These tests confirmed that the only significantly preferred feature in the full sample was for the respirator to be attached using ear-loops (69%) rather than a head-strap (20%) (W = 4160, *P < .001*). No other feature showed a significant preference (.052 ≤ *P* ≤ .92), although a preference for plain (45%) rather than patterned (30%) respirators approached significance (W = 1479, **P* *= .052).

Some reasons given for why the children in the FGDs seemed to prefer respirators with ear-loops rather than head-straps were experience, ease of use and comfort, as illustrated by the comments below.

*We have not used the strap one so we’re not sure about that one, so we like the ear-loop one (FGD6, Grade 3–4, Nepal).*

*I prefer masks with ear-loops because the ones with straps feel so complicated (FGD6, Grade 5–6, Indonesia)*

*I prefer to wear the mask with ear-loops because it is more comfortable (FGD1, Grade 5–6, Indonesia)*

**Fig 3:**
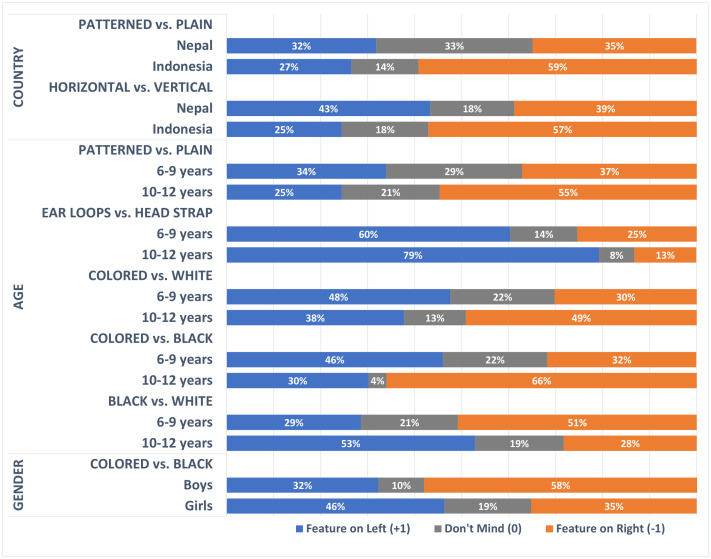
Feature preferences – significant main effects. To aid interpretation: For the main effect of country – patterned vs. plain, the figure shows that plain respirators were preferred by 35% of the children from Nepal and 59% of the children from Indonesia; patterned respirators were preferred by 32% of the children from Nepal and 27% of the children from Indonesia; and 33% of the children from Nepal and 14% of the children from Indonesia did not mind either plain or patterned respirators.

Ordinal regression analyses were conducted to explore whether the country the children came from and demographic variables (i.e., children’s gender, age, and socio-economic status) were predictive of their feature preferences. These analyses, shown in Table 2, provide insights into whether some features were more popular among certain groups of children. The models analyzed the main effects of country (Nepal or Indonesia), gender, age and the parental education level. Univariate analyses indicated significant main effects of country on two features (patterned vs. plain and horizontal-fold vs. vertical-fold); gender on one feature (colored vs. black); and age on four features (patterned vs. plain, ear-loops vs. head-straps, colored vs. black and black vs. white). These main effects are illustrated in Fig 3.

**Table 2:** Ordinal regression models.

Unstandardized coefficient (B)^i^	patterned (+1) vs. plain (−1)	horizontal-fold (+1) vs. vertical-fold (−1)	ear loops (+1) vs head strap (−1)	colored (+1) vs. white (−1)	colored (+1) vs. black (−1)	black (+1) vs. white (−1)
**Univariate models**						
Country (−1)^ii^	−0.726*	−0.787*	0.706^†^	−0.448	−0.127	−0.041
Age^ii^	−0.238*	0.036	0.372**	−0.197^†^	−0.478***	0.240*
Gender (−1)^i^	0.379	−0.205	−0.219	0.554	0.778*	−0.397
Parental education level^iii^	−0.062	−0.161	−0.027	−0.124	0.009	−0.136
**Multivariate models**						
*Main effects*						
Country (−1)	−0.655	−0.856*	0.954^†^	−0.316	−0.396	0.108
Age	−0.236*	0.043	0.348**	−0.221*	−0.594***	0.272*
Gender (−1)	0.147	−0.156	−0.301	0.526	0.783^†^	−0.580
Parental education level	−.0022	−0.037	−0.118	−0.115	0.084	−0.169
*Significant interaction effects* ^iv^						
Country x Age	X	X	X	X	X	X
Country x Gender	−0.420*	X	X	X	0.500*	−0.516**
Country x Parental education level	X	X	X	X	−0.377*	X

^†^*P* < .10, * *P* < .05, ** *P* < .01, *** *P* < .001

^i^The unstandardized coefficients (B) provide an indication of how big the effect sizes are bearing in mind that ratings were obtained on a three-point scale (−1, 0, + 1). For categorical variables like Country (−1), B represents the deviation of the −1 category (i.e., Indonesia) from the grand mean of both categories. For example, B = −0.726 for Country (−1) indicates that the rating for Indonesia was 0.726 lower than the grand mean for both countries (i.e., Indonesian children had a stronger preference for plain respirators). For continuous variables like Age, B represents the increase or reduction in rating for each one-year increase in age. For example, B = −0.238 for Age indicates that the preference for patterned respirators reduced by 0.238 on the three-point scale for each year of age.

^ii^Predictors effect coded (i.e., Country: + 1 = Nepal, −1 = Indonesia; Gender: 1 = Male, −1 = Female)^iii^Predictors mean centered

^iv^Only significant two-way interaction effects are shown in the table (X indicates non-significant effects).

As illustrated in Fig 3, Indonesian children were more likely than the Nepalese children to prefer plain (rather than patterned) respirators and vertical-fold (rather than horizontal-fold) respirators. However, the country effect for the plain (vs. patterned) feature was not significant in the multivariate model when the effects of the children’s age, gender and education of their parents were accounted for.

The effects of age on preferences for the four features that were significant in the univariate models are shown in Fig 3. Patterned (rather than plain) respirators, respirators fixed with head-straps (rather than ear-loops), and colored (rather than black or white) respirators were more popular with younger children than older children. These effects remained significant in the multivariate models.

**Fig 4:**
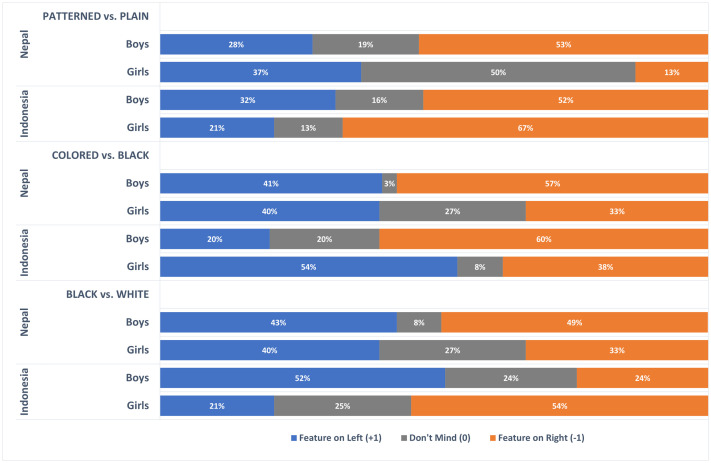
Significant country x gender interaction effects.

Gender significantly predicted preferences for colored vs. black respirators. Boys were more likely than girls to choose black over colored respirators. This main effect was not however significant in the multivariate model, where a significant country x gender interaction was found. Simple ordinal regression analysis on preferences for colored vs. black respirators revealed that gender was significant only in the Indonesian sample (Nepal B = 0.490, *P* = .296, Indonesia B = 1.18, **P* *= .036). Fig 4 shows that, in Indonesia, colored respirators were more popular among girls (54%) than boys (20%), whereas black respirators were more popular among boys (60%) than girls (38%).

The popularity of black respirators among Indonesian boys was also reflected in the significant country x gender interaction for black vs. white preferences (see Fig 4). Simple ordinal regression analysis on preferences for black vs. white respirators indicated that gender was significant only in Indonesia (Nepal B = 0.246, *P* = .595; Indonesia B = −1.36, *P* = .015), where white respirators were more popular among girls (54%) than boys (24%) and black respirators were more popular among boys (52%) than girls (21%).

A significant country x gender interaction was also found for patterned vs. plain preferences. The simple ordinal regression analysis showed that the gender effect on patterned vs. plain preferences was significant only in the Nepalese sample (Nepal B = 1.14, *P* = .017; Indonesia B = −0.604, *P* = .292). Fig 4 shows that, in Nepal, plain respirators were more popular among boys (53%) than girls (13%), while girls were more likely to be indifferent between patterned and plain respirators (50%) compared to boys (19%).

**Fig 5:**
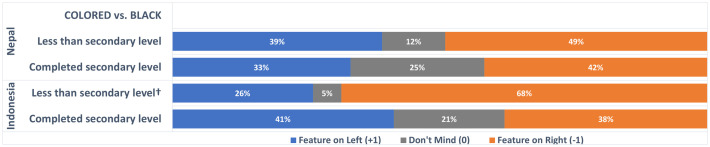
Significant country x parental education level interaction effects.

The final significant interaction was for country x education on colored vs. black preferences. Simple ordinal regression analysis showed that the effect of parents’ education level on preferences for colored over black respirators was significant only in Indonesia (Nepal B = −0.211, *P* = .246; Indonesia B = 0.533, *P = .043*). Fig 5 illustrates that black respirators were more popular than colored respirators (68% vs. 26%) among Indonesian children whose parents had not completed secondary education.

### Why and where children wear masks

When children were asked about why they wear masks, their responses revealed a range of reasons including to shield themselves from allergens and air pollutants such as dust, bad smells, and smoke, as well as from being infected with pathogens.

*There is smoke and dust everywhere, so I wear a mask (FGD5, Grade 2–3, Nepal)*

*I wear it to avoid ‘flu and cough viruses (FGD2, Grade3–4, Indonesia)*

The children also mentioned that they wore masks to prevent them from transmitting infections, such as COVID-19 and the ‘flu, to others.

*I wear a mask when I feel unwell, and I do not want to infect a friend (FGD4, Grade 1–2, Indonesia)*

*To prevent spreading of coronavirus (FGD3, Grade 3–4, Nepal)*

These reasons seemed to be linked to when they began wearing masks, with some stating it was after the start of the COVID-19 pandemic when, in many countries around the world including Nepal and Indonesia, it was mandatory for children and adults to wear masks in public spaces [31,32].

*The first time I wore a mask was in 2020, when COVID happened (FGD6, Grade 5–6, Indonesia)*

*I wore [a mask] after the spread of Corona (FGD3, Grade 3–4, Nepal)*

Notably, some children in Nepal mentioned occasionally wearing masks before the pandemic, but most children began to use them more regularly, during and afterwards. These findings indicate that the COVID-19 pandemic has influenced children’s mask-wearing habits.

*We used to wear it sometimes before COVID-19 but, after, we wore it most of the time (FGD6, Grade 3–4, Nepal)*

The children also mentioned where they usually wear masks. In some responses, the purpose of wearing a mask in particular situations were also stated. The responses suggest that, for some children, mask-wearing had become a regular part of their daily lives and routines.

*I don’t wear masks at home, but I wear them while going out of the house like going shopping (FGD6, Grade 3–4, Nepal)*

*I wear masks for outdoor use: while walking and traveling on motorbike (FGD1, Grade 5–6, Nepal)*

*I always wear [a mask] in school (FGD6, Grade 3–4, Nepal)*

*It’s to avoid dust when riding a motorbike to school (FGD4, Grade 1–2, Indonesia)*

*I wear [a mask] while traveling by bus because there are many people in the bus, and some might have ‘flu, some might be ill, so we wear it to make sure we don’t get a disease from them (FGD6, Grade 3–4, Nepal)*

### Perceptions of mask wearability

It should be noted that the children were referring to the use of any type of face covering/ mask, not specifically respirators. When asked about their perceptions of wearing masks, in two of the Nepal groups (FGD3 and FGD5) all the children said they were comfortable wearing masks. But some of the children in other groups, including the Indonesian groups, did experience some challenges. Difficulty breathing (particularly when playing or running) was raised by several children across a number of groups.

*I don’t feel comfortable because it makes it hard for me to breathe (FGD6, Grade 5–6, Indonesia)*

*In a cloth mask, it is difficult to breathe (FGD2, Grade 2–3, Nepal)*

Issues raised less frequently included sweating around the mouth area, soreness behind the ears, and difficulty talking.

*I feel it’s normal, but it hurts the ears (FGD 2, Grade 3–4, Indonesia)*

*It makes the mouth area sweaty (FGD1, Grade 5–6, Nepal)*

*I got distracted when talking, it’s hard to talk (FGD6, Grade 5–6, Indonesia)*

*Sometimes when the mask is tight, it hurts the back of my ear (FGD 6, Grade 3–4, Nepal)*

## Discussion

The aim of this study was to understand children’s perceptions on wearing respiratory protection, and their preferences for different types and styles of respirators (marketed for children). Comparisons between children from two different countries (Nepal and Indonesia) provides insights into the generalizability of our findings across different cultures, especially across Asia. There have been very few studies on children’s preferences for respiratory protection [19–21], despite the large impact that this may have on their uptake as an intervention, therefore this study provides welcome and important additional evidence.

In the sample as a whole, our findings indicate no dominant or majority preference for specific features, except for respirators fixed using ear-loops (69%) rather than head-straps (20%). This could be explained by supply issues. When we were sourcing respirators to use in this study, we came across very few respirators marketed for children that used head-straps. Most of the respirators we found were fixed to the head using ear-loops. This suggests that the popularity of ear-loops may be driven by familiarity and experience as illustrated by this comment from one of our focus groups:

*We have not used the strap one so not sure about that one, so we like the ear-loop one (FGD6, Grade 3-4, Nepal).*

Ease of donning and doffing of the respirator is another factor that was also picked up by the children in our focus groups and has been suggested by other researchers as contributing to the popularity of respirators utilizing ear-loop strap systems [33,34]. For example, Niu et al. [33] note how respirators designed with two head-straps, which require one strap to be worn over the head and the other behind the neck, can present more challenges for the wearer than respirators with ear-loops. This is illustrated by this comment from one of the children in our focus groups.

*I prefer masks with ear-loops because the ones with straps feel so complicated (FGD6, Grade 5–6, Indonesia)*

In both countries, we also found that colored respirators were more popular with the younger children. Older children were more likely to prefer the white or black respirators. Research supports the idea that color preferences evolve with age. Studies indicate that infants and young children are naturally drawn to bright, saturated colors, while older children and adults tend to prefer more muted or neutral tones [35,36]. This shift is influenced by cognitive development, social conditioning and cultural factors [37]. Additionally, younger children may associate bright colors with playfulness and excitement, whereas older children may favor more neutral tones that align with societal norms of perceived maturity [38].

However, some of the preferences were different between the children from the two countries. Vertical-fold (rather than horizontal-fold) respirators were more popular with the children from Indonesia (57%) than with the children from Nepal (39%). This could be explained by the greater availability of this vertical-fold style of mask in Indonesian shops. Duckbill-style disposable masks (where the filter only covers the part of the mask in front of the nose and mouth), which are similar in appearance to the vertical-fold respirators, are more easily found in Indonesian stores and online shopping platforms than they are in Nepalese stores and online platforms.

It was also notable that gender differences in preferences for either colored, white or black respirators were only found in the Indonesian sample. Black (rather than colored or white) respirators were more popular with the Indonesian boys than the Indonesian girls. This result could reflect adherence to gender norms whereby the color black is associated with traits traditionally linked to masculinity such as power and authority [39]. This explanation does not however explain why the Nepalese boys did not favor the black respirators (over the colored or white respirators) significantly more than the Nepalese girls. We would need to establish that the gender norms and/or black = masculinity association were weaker in the Nepalese sample. Further investigation is also needed to enable more generalized conclusions to be drawn about how the social construction and experience of gender that comes with culture shapes sex differences in color preferences [37,40–43].

The most important take home message is that there is no single design or style of respirator that will appeal to most children. That being said, most of the children had a preference one way or the other for the various features, which suggests that the manufacturers of respirators for children should ideally offer a variety of patterns, colors, and shapes for them to choose from. However, if only one type of respirator can be manufactured (or provided by humanitarian organizations in air pollution crises), plain white or black respirators, fixed using ear-loops, are likely to be more acceptable to most children than patterned, colored and respirators fixed using head-straps. Although patterned or colored respirators may appeal more to younger age-groups, it should be noted that children may be put off by specific types of patterns or colors. As shown in our ranking task, the yellow/gold colored respirator was not popular in our sample. This suggests that children, whatever their age, would probably need to be offered some choice over the specific pattern or color. If governmental agencies or non-governmental organizations plan to recommend, distribute or influence the supply of children’s respirators, taking into account these preferences may support uptake of mask-wearing among children.

In this study, our focus on the designs and styles of respirators is only one element which might influence whether children will wear them consistently. The focus group discussions also highlighted that the wearability and comfort of respirators was important to children. The children in our study identified a number of challenges with mask wearing. For instance, a few mentioned that wearing a mask caused sweating around the mouth area, and made it difficult to breathe while playing, or caused soreness behind their ears. It is therefore important to recognize and address these types of barriers. For example, the ear soreness issue might be resolved by using a respirator with a head-strap or to create a head-strap by using a clip or extender that attaches to the ear-loops. This can also improve the fit of the respirator [44]. The breathability of respirators can also vary and advances in filtration technology can lower breathing resistance, although such respirators tend to be more expensive.

We also gained insights from the focus group discussions into the impact of the COVID-19 pandemic on children’s mask wearing. This was a period when, in Nepal and Indonesia (as well as many other countries around the world), it was mandatory for children and adults to wear masks [45,46]. This started a habit of mask wearing for many of the children in our study. Even though mask wearing was no longer mandatory, it was still part of their lives. Some children said that they were still wearing masks regularly in a range of settings – at school, out shopping, while walking on the street, or riding on a motorbike (with their parents, which is common in both countries). There was also an awareness that wearing masks would not only prevent the spread of diseases but also help to shield them from air pollutants such as dust, bad smells, and smoke. Wearing masks can therefore serve multiple purposes.

Our findings and the conclusions we can draw from them may, of course, be limited to the specific samples of children from Nepal and Indonesia that we recruited to take part. The relatively small sample size and convenience sampling method limit the generalization of our findings, and further research is needed to determine whether the findings are more widely applicable. We have, however, expanded our knowledge of the types of respirators that children prefer. Although our findings may not necessarily generalize to children from other cities or countries, they did show a lot of commonalities in the preferences of the Nepalese and Indonesian children, indicating that they may be transferable, at least to other LMICs with high air pollution levels in Asia.

## Conclusions

This study is the first mixed-method study to analyze children’s preferences for different styles of respirators available in Nepal and Indonesia. Our findings indicate that, while children have varied preferences, respirators with ear-loops are significantly more favored than those with head-straps. Younger children tend to prefer colored and patterned designs, whereas older children lean towards plain and neutral styles. These insights suggest that offering a variety of respirator designs may enhance acceptance and consistent use among children. If only one type of respirator can be manufactured, or distributed as part of a humanitarian effort, plain white or black respirators with ear-loops are likely to be the most broadly accepted. These findings can inform manufacturers and humanitarian agencies aiming to improve uptake of respiratory protection for children in polluted environments. It is important to use resources wisely by providing respirators that children are likely to wear.

## Supporting information

S1 Table Thematic analysis (Indonesia data). (DOCX)

S2 Table Thematic analysis (Nepal data). (DOCX)

S3 Table Demographic characteristics of the children and their carers. (DOCX)

S4 File Inclusivity in Global Research. (DOCX)
