# Supplementary material for: Children’s preferences for features and designs of KN95-style respirators: A comparative study between Indonesia and Nepal
Source: PLoS One. 2025 Oct 17;20(10):e0334116. doi: 10.1371/journal.pone.0334116 (PMC12533843; doi:10.1371/journal.pone.0334116)
Supplement: S1 Table — (DOCX) [file pone.0334116.s001.docx]

**Table S****1** Thematic analysis (Indonesia data)

| **Group** | | 3 | 2 | 1 | 4 | 5 | 6 |
| --- | --- | --- | --- | --- | --- | --- | --- |
| **Type of School** | | Public school | | | Private school | | |
| **Grade of children** | | 1-2 | 3-4 | 5-6 | 1-2 | 3-4 | 5-6 |
| **Theme 1: Mask Use Experience** | | | | | | | |
| **Code** | **Sub-theme: 1.1 When they first started to wear a mask** | | | | | | |
| Code 1 | Wearing a mask since the covid-19 pandemic | - | XX (all) | XX (all) | XX (all) | X (one child) | X (one child) |
| Code 2 | Wearing masks since 2020 | - | X (one child) | X (one child) | X (one child) | X | XX (all) |
| Code 3 | Wearing masks since a certain level of education | XX | - | - | - | X | - |
| Code 4 | Wearing a mask before the covid-19 pandemic | - | - | - | - | X | - |
| Code 5 | Can't remember when they first started wearing a mask | - | - | - | - | X (one child) | - |
| **Code** | **Sub-theme: 1.2 Purpose of wearing a mask** | | | | | | |
| Code 6 | Wearing a mask because of air pollution | - | - | X | - | - | - |
| Code 7 | To avoid the dust | X | X | XX | XX (all) | XX | XX |
| Code 8 | Wearing a mask to avoid smoke | - | - | X (one child) | - | - | - |
| Code 9 | Wearing a mask to avoid the disease in general and specific like Covid-19, influenza or cough | - | X | XX | X | X | X (one child) |
| Code 10 | Wearing a mask when feel unwell so I won’t infect others. | - | X (one child) | X (one child) | - | - | - |
| Code 11 | Wearing a mask without any purpose | - | - | - | - | X (one child) | - |

| **Group** | | 3 | 2 | 1 | 4 | 5 | 6 |
| --- | --- | --- | --- | --- | --- | --- | --- |
| **Theme 2: Where they think they need to wear a mask** | | | | | | | |
| **Code** | **Sub-theme: 2. 1 Place** | | | | | | |
|  | **Sub-Subtheme: 2.1.1 Indoor** | | | | | | |
| Code 12 | Wearing masks at school | XX | XX | XX | XX | XX | XX (all) |
| Code 13 | Wearing a mask inside the house or family/relative’s house | X | - | X (one child) | - | X (on child) | X (one child) |
| Code 14 | Wearing a mask in Quranic course | X | - | X | - | - | - |
| Code 15 | Wearing a mask in the hospital | - | X | - | - | - | - |
| Code 16 | Wearing a mask at the mall | - | - | - | X | X (one child) | X (one child) |
|  | **Sub-Subtheme: 2.1.2 Outdoor** | | | | | | |
| Code 17 | Wear a mask outside the house | X | X | XX (all) | X | X (one child) | X |
| Code 18 | Wearing a mask in the park | - | X (one child) | - | - | X (one child) | - |
|  | **Sub-theme: 2.2. Activity** | | | | | | |
| Code 19 | While playing with friends | - | X | - | - | - | X (one child) |
| Code 20 | Doing anything every time outside | X | XX | XX (all) | X (one child) | X (one child) | XX (all) |
| Code 21 | on Sunday while climbing the mountain | - | - | - | X (one child) | - | - |
| Code 22 | avoid dust when cleaning the house | - | - | - | - | X | - |
| Code 23 | To avoid dust when fishing | - | - | - | X | - | - |
| Code 24 | To avoid dust when on a motorbike | - | - | X | - | X | X (one child) |
| Code 25 | used it when travelling outside | XX | X | X | X | X (one child) | X (one child) |
| Code 26 | I used it when taking out the trash | - | - | - | - | X | - |
| Code 27 | Travel far from home | - | - | X (one child) | - | - | - |
| Code 28 | Go for a walk | XX | X (one child) | - | X | X (one child) | - |

| **Group** | | 3 | 2 | 1 | 4 | 5 | 6 |
| --- | --- | --- | --- | --- | --- | --- | --- |
| **Theme: 3. Consequences of wearing a mask** | | | | | | | |
| **Code** | **Sub-theme: 3.1 Positive Consequences** | | | | | | |
| Code 29 | Feels like normal and still good to breathe | X (one child) | - | X | XX | X (one child) | - |
| Code 30 | Feels comfortable | X | X | X | - | - | - |
| Code 31 | It's hot but feels safe | - | X (one child) | - | - | - | - |
| **Code** | **Sub-theme: 3.2 Negative Consequences** | | | | | | |
| Code 32 | Uncomfortable, stuffy, ear pain, it feels a bit strange | - | X | - | X | - | X |
| Code 33 | Feel disturbed when talking, it’s so difficult | - | - | - | - | - | X |
| Code 34 | Hard to breathe | - | X | - | X | - | - |
| **Theme 4: What children want in masks** | | | | | | | |
| **Code** | **Sub-theme: 4.1 Preferred Features** | | | | | | |
|  | **Sub-subtheme: 4.1.1 Preferred Pattern** | | | | | | |
| Code 35 | Plain mask | - | X | X (one child) | X | XX | X |
| Code 36 | Cartoon figure pattern | X | X | X | - | - | - |
| Code 37 | Flower pattern | - | - | X (one child) | - | - | - |
| Code 38 | Animal pattern | XX | - | X | XX | X | X |
| Code 39 | Indonesian Flag pattern | X (one child) | - | - | - | - | - |
| Code 40 | Rainbow pattern | - | - | X (one child) | - | - | - |
| Code 41 | I like both plain and patterned mask | XX | - | - | - | - | - |
| Code 42 | Ball pattern | - | - | - | - | X (one child) | - |
| Code 43 | Car pattern | - | - | - | - | X (one child) | - |

| **Group** | | 3 | 2 | 1 | 4 | 5 | 6 |
| --- | --- | --- | --- | --- | --- | --- | --- |
|  | **Sub-subtheme: 4.1.2 Preferred Colour** | | | | | | |
| Code 44 | Blue mask | X | X | X (one child) | X (one child) | - | X (one child) |
| Code 45 | Pink mask | X | X (one child) | X (one child) | X | - | - |
| Code 46 | Black mask | X | X | XX | X | X | X |
| Code 47 | White mask | XX | XX | XX | X | XX | XX |
| Code 48 | Red mask | - | - | - | X (one child) | - | - |
| Code 49 | Gold mask | - | - | X (one child) | - | - | - |
| Code 50 | Gray mask | - | X (one child) | X (one child) | - | - | - |
| Code 51 | Brown mask | X (one child) | - | - | - | - | X (one child) |
| Code 52 | Purple mask | X | - | - | X (one child) | - | - |
|  | **Sub-subtheme: 4.1.3 Preferred Shape** | | | | | | |
| Code 53 | Vertical mask | XX (all) | XX (all) | XX | XX | X | X |
| Code 54 | Horizontal mask | - | - | - | X | X | X (one child) |
| Code 55 | Both shape (vertical and horizontal) | - | - | X | - | - | XX |
|  | **Sub-subtheme: 4.1.4 Preferred Strap** | | | | | | |
| Code 56 | Head strap | X (one child) | X (one child) | X (one child) | X | X | - |
| Code 57 | Ear loops | XX | XX | XX | XX | XX | XX |
| Code 58 | Both strap (Head and ear loops) | - | - | X (one child) | X (one child) | X (one child) | - |

| **Group** | | 3 | 2 | 1 | 4 | 5 | 6 |
| --- | --- | --- | --- | --- | --- | --- | --- |
|  | **Sub-theme: 4.2 Easy to use** | | | | | | |
| Code 59 | Easy to wear | X (one child) | - | - | - | - | X (one child) |
| Code 60 | The strap makes it easier to wear with the hijab | - | - | X (one child) | X (one child) | X (one child) | - |
| Code 61 | Simple | X (one child) | - | - | - | - | - |
| Code 62 | Prefer a mask that more suitable with shirt and uniform colour | X | - | - | X (one child) | - | X (one child) |
|  | **Sub-theme: 4.3 Comfortable to wear** | | | | | | |
| Code 63 | I like a mask that comfortable to wear | - | - | - | - | X (one child) | - |
| Code 64 | Choose a mask based on its comfort | - | XX | XX | X (one child) | X | - |

Note: a single X indicated that the code was expressed by 1-4 children or less than half-of the children in a particular group; whereas XX indicated that the code was expressed or agreed upon by 5 or more children, representing half or more of the children in each group.
