# Supplementary material for: Children’s preferences for features and designs of KN95-style respirators: A comparative study between Indonesia and Nepal
Source: PLoS One. 2025 Oct 17;20(10):e0334116. doi: 10.1371/journal.pone.0334116 (PMC12533843; doi:10.1371/journal.pone.0334116)
Supplement: S2 Table — (DOCX) [file pone.0334116.s002.docx]

**Table S2** Thematic analysis (Nepal data)

| **Group** | | 2 | 3 | 1 | 5 | 6 | 4 |
| --- | --- | --- | --- | --- | --- | --- | --- |
| **Type of School** | | Private | | | Public | | |
| **Grade of children** | | 2-3 | 3-4 | 5-6 | 2-3 | 3-4 | 5-6 |
| **Theme 1: Mask Use** | | | | | | | |
| **Code no.** | **Sub-theme 1.1 When they began to wear mask** | | | | | | |
| Code 1 | Started wearing mask before Covid | X | X | XX | X | X | X |
| Code 2 | Started wearing mask after Covid | X | XX | X | X | X | XX |
| Code 3 | Grade they were in when they began to wear mask | X | - | - | - | - | X (one child) |
| Code 4 | I started wearing mask since I was small | X (one child) | - | - | - | - | - |
|  | **Sub-theme 1.2 Reasons for mask use** | | | | | | |
|  | **Sub-sub-theme 1.2.1 Protect from air pollutants** | | | | | | |
| Code 5 | Mask helps to prevent bad smell | X | X | X | - | - | X |
| Code 6 | Mask protects from smoke | - | - | X | X | - | X |
| Code 7 | Mask protects from dust | X | - | X | X | XX | X |
|  | **Sub-sub-theme 1.2.2 Prevent infection transmission** | | | | | | |
| Code 8 | Mask protects from flu | - | X | X | - | - | X |
| Code 9 | Mask protects from Covid virus | X | X | X | X | X | X |
|  | **Sub-sub-theme 1.2.3 Other benefits** | | | | | | |
| Code 10 | Mask make me feel comfortable | X (one child) | - | - | - | - | - |
| Code 11 | People with allergy need to wear a mask | - | - | - | - | - | X (one child) |
|  | **Sub-theme 1.3 Place of mask use** | | | | | | |
|  | **Sub-sub-theme 1.3.1 Use mask while travelling** | | | | | | |
| Code 12 | Wear mask while walking on the street | X | XX | X | - | XX | X |
| Code 13 | Wear mask while travelling on a motor bike | X | - | X | X | - | X |
| Code 14 | Wear mask while travelling in a bus | X | - | - | - | X | - |
| Code 15 | Wear mask while coming to school | - | X | - | - | - | - |

| **Group** | | 2 | 3 | 1 | 5 | 6 | 4 |
| --- | --- | --- | --- | --- | --- | --- | --- |
|  | **Sub-sub-theme 1.3.2 Use mask at school** | | | | | | |
| Code 16 | Wear mask at school | X | X | X | X | - | X |
|  | **Sub-sub-theme 1.3.3 Use mask in crowded space** | | | | | | |
| Code 17 | Wear mask while travelling in a bus | X | - | - | - | X | - |
| Code 18 | Wear mask at school | X | X | X | X | - | X |
|  | **Sub-sub-theme 1.3.4 Use mask during outdoor** **activities** | | | | | | |
| Code 19 | Wear while playing in a park | - | X | - | - | - | - |
| Code 20 | Wear mask while going out of the house (stroll, shopping | X | - | X | X | X | X |
|  | **Sub-theme 1.4 Challenges of wearing mask** | | | | | | |
| Code 21 | No problem with wearing a mask | X | XX (all) | X | XX | XX | XX (all) |
| Code 22 | Wearing mask make back of the ear sore | X | - | - | X | X | - |
| Code 23 | Wearing mask make mouth area sweaty | - | - | X | - | - | - |
| Code 24 | Wearing mask while playing/running make difficult to breathe | X | - | X | - | - | - |
| **Theme 2 Mask preference** | | | | | | | |
|  | **Sub-theme 2.1 Features of mask** | | | | | | |
|  | **Sub-sub theme 2.1.1 Pattern** **preference** | | | | | | |
| Code 25 | Prefer plain mask | - | - | XX | - | XX (6) | XX |
| Code 26 | Prefer patterned mask | XX | X | - | X | X (4) | - |
| Code 27 | Like both patterned and plain/any | - | - | - | - | - | X (one child) |
| Code 28 | Pattern does not matter | - | X | X | - | - | - |
|  | **Sub-sub theme 2.1.2 Colour preference** | | | | | | |
| Code 29 | Like Red colour, Blue (favourite colour) | - | - | X | - | - | X |
| Code 30 | Like Black and White colour | - | - | X | X | XX (half) | - |
| Code 31 | Like White over colourful | - | - | X | - | - | - |
| Code 32 | Like colourful mask | X | - | - | X | XX (half) | - |
| Code 33 | Like Black mask | X | - | - | - | - | X |
| Code 34 | Like White mask | X | - | X | - | - | X |
| Code 35 | Like White over Black | - | XX | - | - | - | - |

| **Group** | | 2 | 3 | 1 | 5 | 6 | 4 |
| --- | --- | --- | --- | --- | --- | --- | --- |
|  | **Sub-sub theme 2.1.3 Shape preference** | | | | | | |
| Code 36 | Prefer any shape/not particular about shape | XX | - | X | XX | XX | - |
| Code 37 | Prefer a horizontal mask | - | - | XX | - | X | - |
| Code 38 | Prefer a vertical mask | - | - | - | - | - | X |
| Code 39 | Mixed preference of shape of mask | - | X | - | - | - | - |
|  | **Sub-sub theme 2.1.4** **Loop/strap preference** | | | | | | |
| Code 40 | Prefer the one with ear loop | X (half) | XX | XX | XX | XX | XX |
| Code 41 | Prefer the one with head strap | X (half) | - | - | X (one child) | - | - |
|  | **Sub-theme 2.2 Most important mask features** | | | | | | |
|  | **Sub-sub theme 2.2.1 Mask that suits** | | | | | | |
| Code 42 | Masks need to look good when we wear | - | - | X (one child) | - | - | - |
|  | **Sub-sub theme 2.2.2 Quality of mask** | | | | | | |
| Code 43 | The one that control pollution more | - | - | - | - | - | X (one child) |
|  | **Sub-sub theme 2.2.3 Colour and pattern matter the most** | | | | | | |
| Code 44 | Black and White | XX | XX | XX | XX | XX | XX |
| Code 45 | Plain and simple mask | - | - | X | - | - | XX |
|  | **Sub-sub theme 2.2.4 Mask with ear loop** | | | | | | |
| Code 46 | Mask with ear loop | - | - | - | - | - | X |

Note: Green boxes or XX refer to half or more responses, blue boxes or X refer to less than half responses and grey colour to no response
