# Supplementary material for: Children’s preferences for features and designs of KN95-style respirators: A comparative study between Indonesia and Nepal
Source: PLoS One. 2025 Oct 17;20(10):e0334116. doi: 10.1371/journal.pone.0334116 (PMC12533843; doi:10.1371/journal.pone.0334116)
Supplement: S3 Table — (DOCX) [file pone.0334116.s003.docx]

**Table S3** Demographic characteristics of the children and their carers

|  | **Full sample (N=116)** | | **Nepal (N=67)** | | **Indonesia (N=49)** | |
| --- | --- | --- | --- | --- | --- | --- |
| **CHILDREN** |  | |  | |  | |
| Gender: Male N (%) | 62 (53.4) | | 37 (55.2) | | 25 (51.0) | |
| Age: Mean (SD) | 9.63 (1.67) | | 9.75 (1.71) | | 9.46 (1.60) | |
| **CARERS** | Carer1^1^ | Carer2^2^ | Carer1 | Carer2 | Carer1 | Carer2 |
| Gender: Male N (%) | 39 (33.6) | 77 (66.4) | 32 (47.8) | 35 (52.2) | 7 (14.3) | 42 (85.7) |
| Age: Mean (SD) | 35.3 (7.7) | 38.2 (6.6) | 32.9 (8.1) | 36.1 (6.7) | 38.4 (5.9) | 40.4 (5.7) |
| **(Nepali) Caste** N (%) |  |  |  |  |  |  |
| Upper caste |  |  | 24 (35.8) | 22 (32.8) |  |  |
| Relatively advantaged Janajati |  |  | 18 (26.9) | 12 (17.9) |  |  |
| Disadvantages Janajati |  |  | 12 (17.9) | 9 (13.4) |  |  |
| Disadvantaged Non-Dalit Janajati |  |  | 2 (2.99) | 2 (2.99) |  |  |
| Religious Minority |  |  | 3 (4.48) | 4 (5.97) |  |  |
| Dalit |  |  | 6 (8.96) | 6 (8.96) |  |  |
| Prefer not to say |  |  | 1 (1.49) | 0 (0) |  |  |
| No response |  |  | 1 (1.49) | 12 (17.9) |  |  |
| **Religion^3^** N (%) |  |  |  |  |  |  |
| Muslim | 50 (43.1) | 47 (40.5) | 2 (2.99) | 3 (4.48) | 48 (97.9) | 44 (89.8) |
| Hindu | 54 (46.6) | 44 (37.9) | 54 (80.6) | 44 (65.7) | 0 (0) | 0 (0) |
| Buddhist | 4 (3.45) | 1 (0.86) | 4 (5.97) | 1 (1.49) | 0 (0) | 0 (0) |
| Christian | 6 (5.17)^4^ | 5 (4.31) | 5 (7.46) | 3 (4.48) | n/a | n/a |
| Catholic | 0 (0) | n/a | n/a | n/a | 0 (0) | 0 (0) |
| Protestant | 0 (0) | n/a | n/a | n/a | 1^4^ (2.04) | 0 (0) |
| Kirat | 1 (0.86) | 1 (0.86) | 1 (1.49) | 1 (1.49) | n/a | n/a |
| Prefer not to say | 0 (0) | 0 (0) | 0 (0) | 0 (0) | 0 (0) | 0 (0) |
| No response | 1 (0.86) | 18 (15.5) | 1 (1.49) | 13 (19.4) | 0 (0) | 5 (10.2) |
| **Education level of carers**  **N (%)** |  |  |  |  |  |  |
| No formal schooling | 0 (0) | 3 (2.59) | 0 (0) | 3 (4.48) | 0 (0) | 0 (0) |
| Some primary school (Nepal: grade 1-5; Indonesia: 1-6) | 5 (4.31) | 9 (7.76) | 2 (2.99) | 8 (11.9) | 0 (0) | 1 (2.04) |
| Completed primary/ elementary school (Nepal: grade 1-5; Indonesia: 1-6) | 5 (4.31) | 4 (3.45) | 5 (7.46) | 3 (4.48) | 0 (0) | 1 (2.04) |
| Completed middle/ junior high school (Nepal: grade 6-8; Indonesia: 7-9) | 15 (12.9) | 12 (10.3) | 12 (17.9) | 11 (16.4) | 3 (6.12) | 1 (2.04) |
| Completed secondary/ higher secondary school (Nepal: grade 9-12; Indonesia: 10-12) | 41 (35.3) | 35 (30.2) | 25 (37.3) | 18 (26.9) | 16 (32.6) | 17 (34.69) |
| Completed post-secondary/ vocational training but degree level | 14 (12.1) | 11 (9.48) | 9 (13.4) | 4 (5.97) | 5 (10.2) | 7 (14.28) |
| Completed University bachelor's degree/ other degree-level qualification | 22 (18.9) | 15 (12.9) | 3 (4.48) | 1 (1.49) | 19 (38.8) | 14 (28.57) |
| Completed University postgraduate degree | 4 (3.45) | 4 (3.45) | 2 (2.99) | 2 (2.99) | 8 (16.3) | 2 (4.08) |
| Prefer not to say | 7 (6.03) | 5 (4.31) | 4 (5.97) | 4 (5.97) | 3 (6.12) | 1 (2.04) |
| No response | 2 (1.72) | 18 (15.52) | 2 (2.99) | 13 (19.40) | 0 (0) | 5 (10.20) |

| Table S1 cont… |  |  |  |
| --- | --- | --- | --- |
|  | **Full sample (N=116)** | **Nepal (N=67)** | **Indonesia (N=49)** |
| **Household income**^5^ |  |  |  |
| Nepalese NPR |  |  |  |
| Min-Max NPR |  | 2,000 – 240.000 |  |
| Mean (SD) NPR |  | 36,045 (38,814) |  |
| No response |  | 23 (34%) |  |
| Indonesian IDR N (%) |  |  |  |
| IDR ≤1.5 million |  |  | 2 (4.06) |
| IDR 1.5-2 million |  |  | 8 (16.3) |
| IDR 2-5 million |  |  | 13 (26.5) |
| IDR 5-24 million |  |  | 11 (22.4) |
| IDR ≥ 24 million |  |  | 1 (2.04) |
| No response |  |  | 14 (28.6) |

*Notes:* **^1^**Carer 1 is the carer who provided consent for the child to take part in the study. **^2^**Carer 2 is Carer 1’s spouse/partner. **^3^**Options provided to participants were different in Nepal and Indonesia. **^4^**Protestant (N=1) was counted as Christian. **^5^**Options provided to participants were different in Nepal and Indonesia.
